# Rhino-orbital Mucormycosis: Clinical Findings and Treatment Outcomes of Four Cases

**DOI:** 10.4274/tjo.82474

**Published:** 2015-08-05

**Authors:** Şeyda Karadeniz Uğurlu, Sedat Selim, Aylin Kopar, Murat Songu

**Affiliations:** 1 Katip Çelebi University Faculty of Medicine, Department of Ophthalmology, İzmir, Turkey; 2 Katip Çelebi University Faculty of Medicine, Atatürk Teaching and Research Hospital, Clinic of Ear-Nose and Throat, İzmir, Turkey

**Keywords:** Mucormycosis, orbital involvement, exenteration

## Abstract

In this case report, we present the clinical findings and therapeutic outcomes of four rhino-orbital mucormycosis patients. The four patients (1 female, 3 male; age range, 55-77 years) all had diabetes mellitus and two also had chronic renal failure. All patients exhibited proptosis, sinusitis, and dark-colored lesions on the nasopharynx and/or hard palate; three patients had ipsilateral peripheral facial paralysis. Visual acuity was no light perception in the two patients with severe orbital involvement and 0.8 in two patients with limited orbital involvement. Histopathological examination of the hard palate, nasopharynx or sinus biopsy revealed typical Mucor hyphae. Systemic liposomal amphotericin B was initiated in all patients. The patients with limited ocular involvement received amphotericin B both intravenously and by local irrigation; both patients had complete recovery. The other two patients underwent orbital exenteration; one patient died after declining systemic treatment postoperatively. Rapid diagnosis and treatment are important for the survival of rhino-orbital mucormycosis patients. With orbital involvement, surgical debridement and systemic and local treatment with antifungal agents may help avoid mutilating surgery like exenteration.

## INTRODUCTION

Mucormycosis is a rapidly progressing fungal infection caused by filamentous fungi in the Mucoraceae family and is frequently seen in diabetic and immunocompromised patients. Mucormycosis is categorized as rhinocerebral, pulmonary, cutaneous, gastrointestinal or disseminated, depending on organ involvement; the most common form is rhinocerebral (39%).^1^ This form may be divided into subtypes based on which tissues are affected: rhinonasal, rhinoorbital or rhinoorbitocerebral.

Rhinoorbital infection begins when fungal spores are inhaled and invade the nasal mucosa, and sinusitis develops as the fungus spreads to the paranasal sinuses. Orbital involvement occurs when the infection invades the orbital wall from the paranasal sinuses. Symptoms may include pain, chemosis, vision loss, ophthalmoplegia and proptosis. Ophthalmoplegia arises from infection of the muscles and orbital space or when the third, fourth and sixth cranial nerves are affected. Peripheral seventh cranial nerve paresis or paralysis and hypoesthesia of the face are often observed.^1,2^

This study presents the clinical findings and treatment outcomes of four patients with orbital mucormycosis following nasal involvement.

## CASE REPORTS

### Case 1

**Figure 1:**
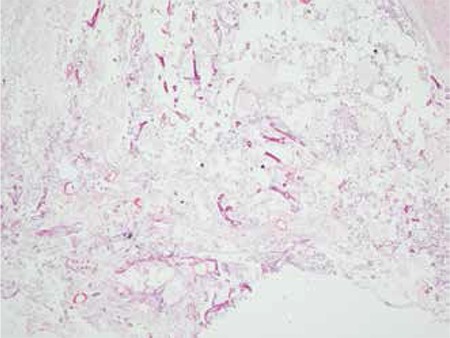
Case 1, palate biopsy showing thick-walled, large diameter (6-25 micron), nonparallel branching hyphae typical of mucormycosis (Hematoxylin&eosin stained)

**Figure 2:**
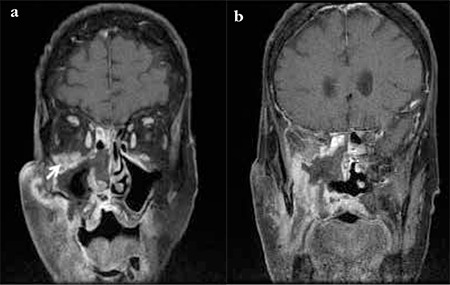
Case 1, infiltration of the lower side of the orbit (a, white arrow) and probable infiltration of the apex and dura through the inferior orbital fissure (b)

A 75-year-old female diabetic patient presented with complaints of swelling of the right cheek and eye. Upon examination, a 2x2 cm crusted lesion was found on the right hard palate. On ophthalmologic examination, visual acuity was 8/10 in the right eye and 10/10 in the left eye. Proptosis (20/17 mm) and lateral gaze limitation were observed in the right eye, and there was facial paralysis of the right side. Cranial and orbital magnetic resonance imaging (MRI) with contrast revealed widespread inflammatory changes primarily suggesting a fungal infection extending to the premaxillary soft tissue of the right maxillary sinus, infratemporal and pterygopalatine fossa, and the parapharyngeal area; multi-level abscess images were observed. The abscessiform lesion also penetrated into the lateral wall of the right orbit. Material biopsied from the palate was histopathologically consistent with mucormycosis (Figure 1). Treatment with 3 mg/kg/day liposomal amphotericin B was initiated. Additionally, right endoscopic medial maxillectomy and irrigation with liposomal amphotericin B were performed. MRI on postoperative day 3 showed infiltration of the orbit, inferolateral area and orbital apex via the inferior fissure; infiltration of the dura was also suspected (Figure 2). The possibility of conducting a second, aggressive surgical procedure was considered by a council of physicians from related disciplines, but the patient’s family refused a second surgical intervention. Therefore, it was decided to continue treatment with local irrigation of the orbit and sinus cavity with amphotericin B via drainage line, and the patient was monitored with MRI. Follow-up orbital and cranial MRI images showed regression of the lesion. After receiving liposomal amphotericin B for 34 days, oral posaconazole (400 mg twice daily) was started and the patient was discharged from the hospital. Follow-up 3 months later showed reduced proptosis, free movement of the eye in all directions and ophthalmologic examination was normal.

### Case 2

**Figure 3:**
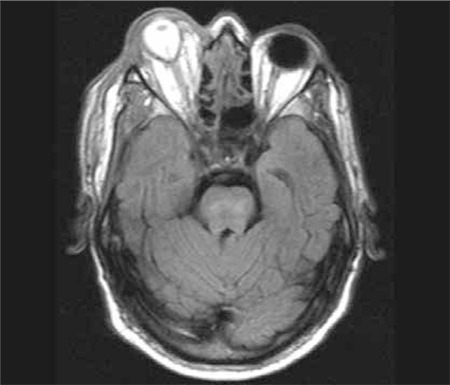
Case 2, infiltration of the right retroorbital tissue, severe proptosis and conical deformation of the posterior globe

A 63-year-old male patient presented with pain in the right eye, redness, swelling and loss of sensation in the right cheek that had developed one month earlier. Examination revealed right facial paralysis and necrotic crust and lesions on the hard palate and nasal passages, and the patient was admitted with a primary diagnosis of mucormycosis. It was learned that the patient had diabetes mellitus (DM) and had begun hemodialysis due to chronic renal failure. Surgical debridement of the area was conducted in the ENT clinic and the material was sent for pathologic analysis. The patient’s right eye displayed severe proptosis, eyelid edema and limited movement in all directions. Visual acuity was at the level of no light perception in the right eye and 5/10 in the left eye. Slit-lamp examination revealed severe chemosis and corneal opacification in the right eye. Contrast MRI showed infiltration of the optic nerve and retroorbital tissue creating exophthalmos of the right globe. The right globe had assumed a conical shape due to outward pressure (Figure 3). Histopathological diagnosis confirmed mucormycosis and treatment with 3 mg/kg/day liposomal amphotericin B was initiated. After acquiring informed consent from the patient, exenteration was performed on the right eye (Figure 4a and 4b). On the ninth day of liposomal amphotericin treatment, the patient refused further treatment and was discharged at his own request. It was learned that the patient died six months later.

### Case 3

**Figure 5:**
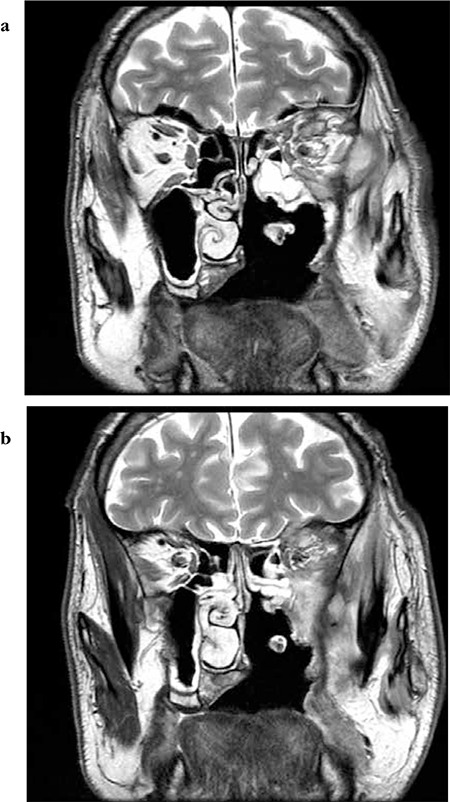
(a and b) Case 3, coronal magnetic resonance imaging images of the orbit showing increased signal intensity of both intraconal and extraconal fat, edematous thickening of the recti and orbital apex infiltrationa

**Figure 6:**
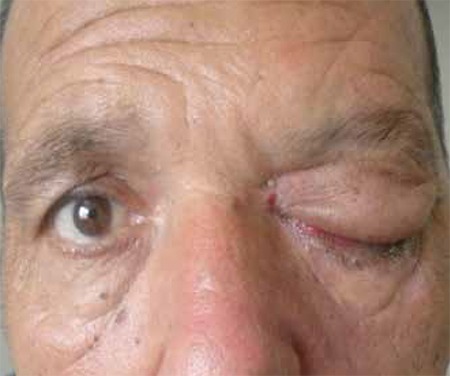
Case 3, patient exhibiting marked proptosis and total ophthalmoplegia of the left eye

A 59-year-old male patient presented with complaints of ptosis and edema of the left eyelid and facial pain beginning 9 days earlier. The patient had a 15 year history of DM and hypertension, and had undergone treatment for chronic renal failure for the past year. Examination in the ENT clinic revealed necrotic tissue in the left nasal passage and hard palate, and there was left facial paralysis. Paranasal sinus CT showed a lobulated, soft mass causing destruction in the left maxillary, ethmoid and sphenoid sinuses. Orbital MRI showed invasion of the mass into the left orbit and erosion of the lateral, medial and inferior orbital walls (Figure 5). On ophthalmologic examination, the left eye had no light perception, proptosis and limited eye movement in all directions (Figure 6). Sinus surgery and debridement were performed by the ENT clinic and a biopsy was taken. After confirming the mucormycosis diagnosis, 3 mg/kg/day liposomal amphotericin B was started and exenteration was conducted on the left side. During follow-up, debridement was repeated and amphotericin B irrigation was performed to facilitate socket healing.

### Case 4

**Figure 7:**
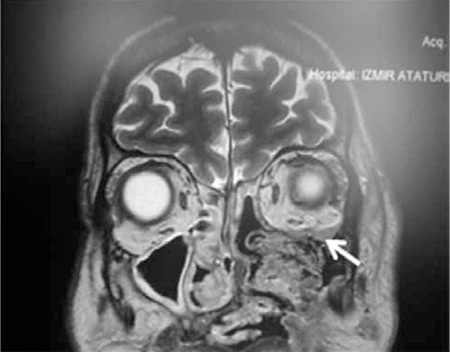
Case 4, magnetic resonance imaging images of patient number 4 who underwent left maxillectomy and hard palate resection. The area of suspected involvement in the inferior wall orbital wall is shown with a white arrow

**Figure 8:**
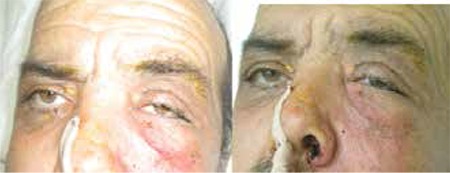
Case 4, patient preoperatively (a) and on postoperative day 2 after surgical debridement and amphotericin B irrigation (b). Note that there is marked improvement in eyelid edema and hyperemia

A 57-year-old male patient presented to our hospital after one month of sinusitis treatment failed to alleviate his complaints of facial pain and swelling. The patient’s medical history included DM. Examination showed periorbital edema and ptosis of the left eye and hyperemic induration of the left cheek area including the upper lip and left labial commissure. Necrotic, black ulceration was observed over most of the soft and hard palate. Visual acuity was 10/10 on the right and 8/10 on the left. The right eye appeared normal, while the left upper and lower eyelids exhibited hyperemia and edema. Eye movement was free and painless. Pupillary light reflexes were bilaterally positive, Marcus Gunn negative. Optic nerve and ocular muscles appeared normal in paranasal CT and facial MRI, but an area of suspicious involvement was seen in the inferior orbital wall (Figure 7). By recommendation of the Infectious Diseases Clinic, treatment with 4.5 g intravenous piperacillin/tazobactam three times a day, 500 mg metronidazole twice a day, and 3 mg/kg/day amphotericin B was initiated. Left Caldwell-Luc procedure, endoscopic medial maxillectomy and hard palate resection were conducted. Ophthalmologists observed no major orbital invasion during preoperative evaluation. The pathology report confirmed mucormycosis diagnosis and systemic treatment was continued. One week later, debridement was repeated and lavage with amphotericin B was performed. Two days later, induration of the left lower eyelid showed clinical regression (Figure 8a and 8b). Chemosis resolved by the end of the first postoperative week. Treatment with systemic liposomal amphotericin B and local maxillary sinus irrigation continued. The patient was discharged with oral posaconazole (400 mg twice daily) following amphotericin B treatment.

## DISCUSSION

Mucormycosis is a condition with a fulminant course and a high mortality risk. The most common predisposing factor is DM (60-80%), though hematologic diseases, neoplasias, chronic renal failure, antineoplastic agents, immunosuppressive therapy, corticosteroid use, protein-calorie malnutrition, organ and bone marrow transplantation, and other conditions resulting in immunosuppression such as AIDS also factor in its etiology.^3^ In a study by Yohai et al.^2^ including 145 cases, the most common predisposing factor was DM (60%). Similarly, Ferry et al.^4^ found DM to be the greatest predisposing factor in their study population, with 83%. Gumral et al.^5^ reviewed reports in the Turkish literature between 2000 and 2010 and found that among 79 cases of mucormycosis, the predisposing factor was diabetes in 32 cases and hematologic pathologies in 32 other cases. Another risk factor for mucormycosis infection is renal failure. In these patients, acidosis as well as chronic desferoxamine use contributes to the progression of zygomycosis.^6,7^ In our population, all patients had DM. Two patients also had chronic renal failure; the course of mucormycosis in these two patients was more severe and required exenteration due to extensive orbital invasion.

With orbital involvement, clinical symptoms and findings include periorbital edema, pain, proptosis, ophthalmoplegia and decreased vision. Yohai et al.^2^ reported periorbital edema in 43% of their patients, periorbital pain in 11%, proptosis in 64%, ophthalmoplegia in 67% and vision impairment in 65%. In the same study, 22% of patients had facial paralysis and 20% had facial hypoesthesia. Among the 4 patients in our study, 2 had severe vision loss, proptosis and frozen orbit, and restricted eye movement was observed in another patient. Facial nerve involvement and facial hypoesthesia were found in 3 patients.

The early diagnosis and treatment of mucormycosis is very important in terms of prognosis. Patients who begin treatment within 6 days have a survival rate of 76-81%, while a treatment delay of more than 12 days reduces this rate to 36-42%.^8,9^ In our study, the time between symptom onset and hospital admission varied between 9 days and 1 month. Compared with the literature, these patients were late in seeking medical help and this delayed the initiation of treatment.

Amphotericin B has become the gold standard in the systemic treatment of mucormycosis. Before the use of amphotericin B, the survival rate of mucormycosis patients was just 6%, whereas after the introduction of amphotericin B this rate dramatically increased to the 60% range.^10,11,12^ Liposomal amphotericin B is the first choice of treatment, as it crosses the blood-brain barrier more effectively.^11,13^ Another drug that can be used in systemic treatment is posaconazole. In a large European study, fluconazole was ineffective, itraconazole was found to be partially effective, and posaconazole was effective.^14,15,16,17,18^ For our patients, oral posaconazole was chosen as maintenance antifungal therapy after the completion of amphotericin B treatment.

A critical stage in the treatment of mucormycosis is debridement of necrotic tissue. In our study, all cases underwent surgical debridement of necrotic areas; endoscopic maxillectomy and hard palate resection were added when deemed necessary. Two patients with extensive orbital mucormycosis invasion and total vision loss underwent exenteration. Exenteration was deemed necessary to reduce the fungal load and prevent cerebral involvement and the fatal progression of the disease. Despite extension to the orbit in the other two patients, close monitoring, medical therapy and local amphotericin B irrigation were preferred due to good visual potential and positive response to treatment.

As mucormycosis forms vascular obstruction, it may be difficult for drugs to reach the affected tissues. For this reason, local irrigation of infected areas with amphotericin B is used in conjunction with surgery and systemic therapy. Seiff et al.^19^ reported that among 7 cases of mucormycosis treated with local amphotericin B irrigation in addition to surgery and systemic therapy, the fungal progression stopped or regressed in 6 patients, and only 1 patient required exenteration. In a multi-center study, Kaya et al.^20^ used local amphotericin B irrigation together with different standard treatments in 4 patients, but all of those patients died. Songu et al.^21^ reported that in 3 cases that underwent debridement and local amphotericin B irrigation in addition to systemic treatment, spread of the fungal infection was successfully halted and none of the cases required exenteration. Konuk et al.^22^ reported 2 fatal cases that did not receive amphotericin B irrigation, whereas in a report of 2 non-fatal cases by Özay et al.,^23^ one patient received local irrigation and the other did not. In our study, 3 out of 4 patients underwent local amphotericin B irrigation. Local irrigation was effective in facilitating socket healing in one patient following exenteration, while in the other two patients, irrigation was used in the maxillary sinus pathways and contributed to the healing of their orbital infection.

Orbital involvement in a high-mortality infection like mucormycosis may necessitate the decision to perform exenteration. This difficult decision is made when the extent of the disease and the risk of mortality outweigh the desire to keep the patient’s globe in place. Local antifungal therapy in the form of irrigation can be used in addition to systemic treatment and debridement to save the globe. Local use of antifungal agents facilitates their delivery to affected tissues. Patients’ general condition, course of the disease and response to treatment should be closely monitored, and exenteration should be performed if there is life-threatening progression of the disease.

## Figures and Tables

**Figure 4a:**
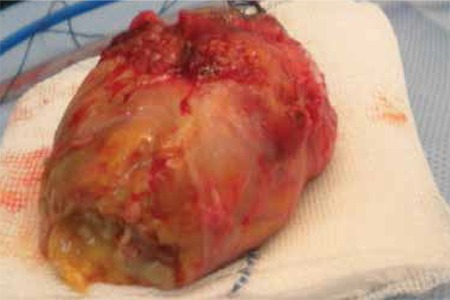
Case 2, exenteration material

**Figure 4b:**
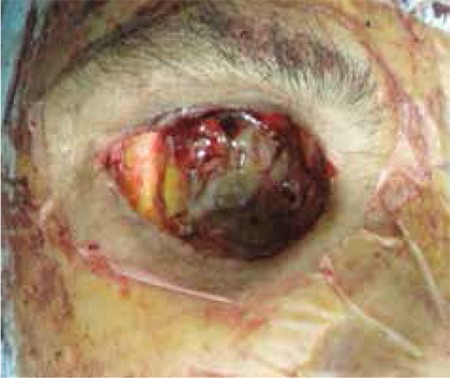
Case 2, necrotic changes in the socket and continued suppuration at the apex are visible following exenteration
